# Socially assistive robotics for post-stroke rehabilitation

**DOI:** 10.1186/1743-0003-4-5

**Published:** 2007-02-19

**Authors:** Maja J Matarić, Jon Eriksson, David J Feil-Seifer, Carolee J Winstein

**Affiliations:** 1 Computer Science Department, University of Southern California, Los Angeles, CA, USA; 2 Department of Neurology, University of Southern California, Los Angeles, CA, USA

## Abstract

**Background:**

Although there is a great deal of success in rehabilitative robotics applied to patient recovery post stroke, most of the research to date has dealt with providing physical assistance. However, new rehabilitation studies support the theory that not all therapy need be hands-on. We describe a new area, called *socially assistive robotics*, that focuses on non-contact patient/user assistance. We demonstrate the approach with an implemented and tested post-stroke recovery robot and discuss its potential for effectiveness.

**Results:**

We describe a pilot study involving an autonomous assistive mobile robot that aids stroke patient rehabilitation by providing monitoring, encouragement, and reminders. The robot navigates autonomously, monitors the patient's arm activity, and helps the patient remember to follow a rehabilitation program. We also show preliminary results from a follow-up study that focused on the role of robot physical embodiment in a rehabilitation context.

**Conclusion:**

We outline and discuss future experimental designs and factors toward the development of effective socially assistive post-stroke rehabilitation robots.

## Background

Stroke is a major cause of neurological disability. Most of those affected are left with some loss of movement. Through concerted use and training of the affected limb during the critical post-stroke period, such disability can be significantly reduced [1]. The rate and amount of recovery greatly depends on the amount of focused training, along with stroke severity and cognitive availability. Evidence shows that the intensity and frequency of focused therapy can improve functional outcomes [2]. However, since such rehabilitation normally requires supervision of trained professionals, lack of resources limits the amount of time available for supervised rehabilitation. As a result, the quality of life of patients post stroke is dramatically reduced, and medical costs and lost productivity continue to be incurred.

Due to the high instance of stroke today, and its increasing rate in the growing elderly population, post-stroke robot-assisted therapy is an area of active research. A number of effective systems have been developed, using physical assistance in order to achieve rehabilitative goals. However, not all effective rehabilitation therapy requires the use physical contact between the therapist and the patient. Non-contact therapy constitutes the motivation for our work on robotic social interaction as a tool for post-stroke rehabilitation. In this paper, we describe a contact-free socially-assistive post-stroke therapeutic robot system. We also show how such therapy methodology fits into currently used post stroke therapies, since the goal of socially assistive robots is not to replace existing therapies and therapists but to augment current options and allow for greater flexibility for both patients and therapists.

### Post-Stroke Assistive Robotics

Robotics used for post-stroke rehabilitation falls under the broad field of Assistive Robotics (AR). This research area includes rehabilitative robotics, wheelchair robots and other mobility aids, companion robots, manipulator arms for the physically disabled, and educational robots. Assistive robots are intended for use in a range of environments, including hospitals, physical therapy centers, schools and eventually homes. As noted earlier, the vast majority of post-stroke rehabilitative robots rely on physical interaction to achieve their therapeutic goals [3-6]. However, physical contact between a robot and a patient and, in some cases, the powered movement of a patient's arm by a robot, creates legitimate safety concerns. Such concerns can be prohibitive in introducing a novel therapy.

### Constraint-Induced Therapy

In addition to safety issues involved in hands-on machine assisted rehabilitation, other reasons exist for considering hands-off non-contact rehabilitation as a complement, not a replacement, for hands-on therapy. Specifically, effective therapeutic regimens involving no physical contact between the therapist and patient have been demonstrated. As a prominent example, Constraint-Induced (CI) therapy is a method currently undergoing Phase III evaluation [7] that has promise (results under review) to increase upper-limb functionality [8] when performed during the initial plasticity period following a stroke and up to a year after [9].

The therapy requires the patient to wear a constraining mitt over the arm unaffected by stroke for the fourteen waking hours of the day. During this period, the patient is asked to do as much as physically possible with the stroke-affected arm in order to promote recovery and re-learning. For up to six hours per day, the patient undergoes physical therapy of a functional nature. During this time, the patient is asked to perform several daily tasks, such as moving pencils from one bin to another, turning pages in a newspaper, and putting magazines on a shelf. The patient is monitored by a physical therapist and given encouragement and verbal suggestions as to the proper muscle movements; however, no physical assistance is given.

CI therapy has been shown to be effective, but it requires many hours of dedicated one-on-one care between the patient and the physical therapist. Given the vast and growing post-stroke population, the availability of an individual therapist for up to six hours per day is not practical. The resulting need creates a niche for *socially assistive robotics *technology capable of filling the gap created by the lack of availability of human care.

Furthermore, studies have shown that the major cause of poor adherence to and lack of compliance with rehabilitation exercises is due to a lack of motivation [10]. A personalized robot can monitor progress during the physical therapy and daily life, and provide tireless motivation, encouragement, and guidance to the patient, without involving any physical contact. Such socially assistive technology is the focus of our work described in this paper.

### Socially Assistive Robotics

Fong et al. [11] described a taxonomy for Socially Interactive Robots (SIR), machines that interact primarily through social interaction. The term was coined in order to distinguish tele-operation (i.e., remote control) from social interaction in Human-Robot Interaction (HRI). We define *Socially Assistive Robotics (SAR) *as the intersection of Assistive Robotics (AR) and SIR [12]. SAR shares with AR the goal of providing assistance to human users, but SAR constrains that assistance to be through non-physical social interaction. Rather than focus on the interaction itself, as is done in SIR, SAR focuses on achieving specific convalescence, rehabilitation, training, or education goals. By addressing social rather than physical interaction, the majority of the safety concern is alleviated. The motivation for defining this new and growing research area comes in response to a new niche in rehabilitation, which can bring together researchers from multiple disciplines around a promising new area of research.

### Taxonomic Description

In Fong et al. [11], several relevant concerns and methods of classifications were collected into the following taxonomy (see Table 1) for classifying socially interactive robotics (SIR).

To create a taxonomy of SAR, we include all of the elements from the above SIR taxonomy, and also add the following new components (see Table 2) specific to SAR [12].

**Table 1:** 

Embodiment	Representing an abstraction as a physical entity.

Emotion	Impulse that moves an organism to action.

Dialog	Joint process of communication.

Personality	The set of distinctive qualities that distinguish individuals.

Human-Oriented Perception	The ability to perceive the world as humans do. Relating those perceptions in human-understandable terms.

User Modeling	The ability to measure human social behavior. The interpretation of human behavior.

Socially Situated Learning	An individual acting with its social environment to acquire new competencies.

Intentionality	Individual's actions are a result of intended behaviors by the individual.

**Table 2:** 

User Populations	The populations that the robot is meant to interact with. Examples include the elderly, individuals with physical impairments, individuals in convalescent care, individuals with cognitive disorders, and students.
Task Description	The task that the robot is meant to achieve. Examples include tutoring, physical therapy, and daily life assistance.

Sophistication of Interaction	How the robot interacts with a user, and how the user in turn reacts to the robot.

Role of Robot	Is the robot a physical therapist, a nurse's assistant, a tutor, etc.?

In the research presented in this paper, we focus on the above components of the taxonomy: embodiment, personality, user modeling, the task description, and the role of the robot in the rehabilitation process.

Each is briefly discussed next.

#### Embodiment

The role of the physically embodied robot in a socially-assistive context is of key importance, yet may be seen as counter-intuitive. It may not be obvious why a robot is needed at all, when instead a personal digital assistant (PDA) or ubiquitous home computer system might be used. While there is ample anecdotal evidence to support the importance of the physical robot sharing the context of the user and its positive impact on user engagement and motivation, there are currently few concrete results that compare robots to computers and other assistive technologies. This is therefore one of the foci of our research.

#### Personality

The personality of a robot could have great effect on the patient's compliance with and enjoyment of that robot. One study has addressed the effects of personality on a user's performance during a task [13], however there has so far been no work on the long-term effects of the robot's personality on the effectiveness in a rehabilitation task. Since it has been shown that pre-stroke personality has an impact on the rate of post-stroke recovery [14], it is important to explore how user personality relates to robot personality in rehabilitative settings. We have so far performed two studies focused on user personality, the use of personal space, and user-robot personality matching [15,16]. Many challenging research issues remain to be addressed in order to gain insight into time-extended personalized socially assistive human-robot interaction.

#### User Modeling

Another area of relevant research is how to effectively observe and model the patient in a therapeutic setting. In addition to monitoring task performance, it is also important for the robot to observe the patient's social affect indicated by facial expressions, gestures, tone of voice, and body language. Our work is also beginning to measure therapy-relevant signals such as interest and frustration levels. These factors would directly inform the system of the condition of the patient and allow the robot to modulate its interaction in order to maximize its effectiveness. The use of such physiological signals as input for robot-assisted post-stroke therapy is a new and promising direction of assistive robotics.

#### Task Description and Role of the Robot

The description of the task and the role of the robot have great influence on each other and the process of robot-assisted therapy. Since we intend to insert a robot aide into an existing therapy regimen, the robot's goals must not interfere with or needlessly duplicate existing efforts made during therapy. Instead, robot-assisted therapy must complement existing care to enhance the experience for the patient. Because time-extended interactions with the patient, involving many hours per day and for several months, issues of robot personality, authority, and attachment must also be considered.

This paper outlines our pilot work addressing the key issues above, and describes ongoing follow-up work that continues to expand on those results toward increased effectiveness of socially assistive robotics for recovery post-stroke.

## Methods and Results

A key goal of our research is to gain insight into assistive human-robot interaction (HRI). Toward that end, we have performed pilot studies examining the effects of HRI modalities on post-stroke therapy performance, and methods for user modeling involving motion capture. This section describes those studies. We performed a pilot study with a socially assistive mobile robot. This robot participated in simple therapeutic interactions with patients post-stroke in the process of performing rehabilitation exercises such as arm movements and shelving magazines [17]. The approach involved the development of a safe, user-friendly, and affordable mobile robot, capable of following the patient in an indoor environment. The robot monitored the patient's use of the stroke-affected limb, and provided encouragement, guidance, and reminders. It also logged the patient's movement of the affected limb and kept track of rehabilitation progress for reporting to the physical therapist.

The robot behaved in response to the sensed movements of the monitored stroke-affected limb. It provided gentle reminders and prompting to the participant if the affected arm had not been active for some period, and praise and encouragement if it had. The robot was also able to report performance data in analytical form to the rehabilitation staff, for use in fine-tuning the robot-assisted therapy.

### Motion Capture

**Figure 1:**
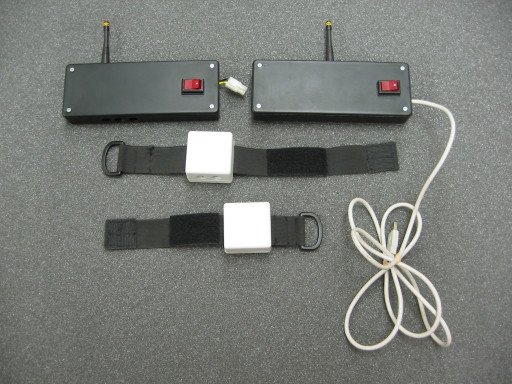
**Motion capture mechanism**. Components of the motion capture mechanism used in monitoring limb movement. Shown are the transmitter and receiving units (top left and right) and two of the sensor arm-bands (bottom and middle). For scale, sensor box (white) on the arm-band is 5.5 cm on the side.

**Figure 2:**
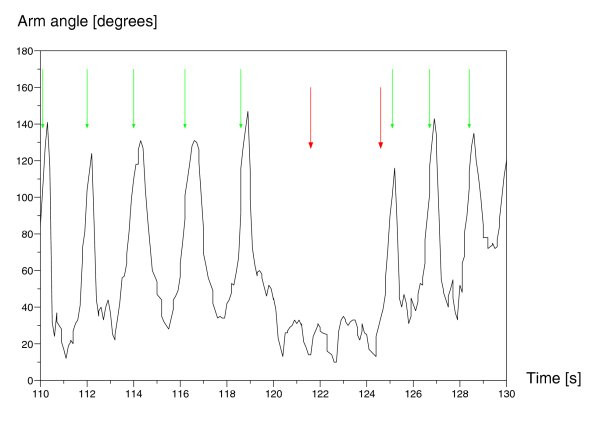
**Motion capture output**. A patient's arm activity during an experiment. The thin arrows show when a reaching motion is detected. The thick arrows show when arm inactivity triggers the robot to encourage the patient.

Monitoring a patient's progress during a task is of paramount importance to effective therapy. Jovanov et al. [18] have shown that computer monitoring of a patient's progress in a walking task can be effectively employed for computer-assisted therapy. We developed a portable motion capture system (see Figure 1) which registers the patient's movement with light-weight inertial measurement units (IMU) worn on the monitored limb, much like a wristwatch on a Velcro strap [19]. Data from the motion capture units (see Figure 2) are sent wirelessly to a receiver on the robot for analysis, thus providing the robot with real-time and accurate patient movement monitoring capability. Importantly, this mechanism does not require the patient to sit or stand in a particular area; the patient can move freely both indoors and outdoors, thereby providing feedback about natural functional movements as well as specific rehabilitation exercises.

We conducted a pilot study on the effectiveness of using motion capture and non-contact reinforcement with 6 subjects that are part of a larger IRB-approved stroke rehabilitation study. Each participant was monitored using the above-described motion capture system. Only a single capture unit was used on the affected limb, as only up-down movement was used in this study; the use of multiple units would provide more detailed information about limb use.

### Design

**Figure 3:**
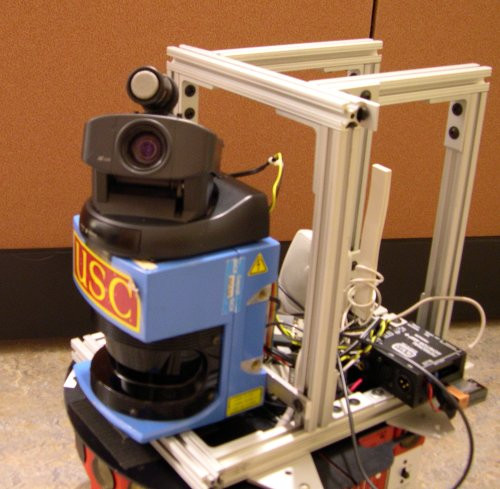
**Pioneer mobile robot**. The mobile robot base used in the experiments. Shown is the laser (box at bottom with USC sticker), camera (mounted on top of the laser), and microphone (mounted on top of the camera).

The robot (see Figure 3) used a standard Pioneer2 DX mobile robot base. A SICK LMS200 scanning laser range-finder enabled it to find and track the participant's legs. For obstacle avoidance, the laser is used together with the on-board sonar array. A Sony pan-tilt-zoom (PTZ) camera allows the robot to "look" at and away from the participant, shake its "head" (camera), and make other communicative actions. The camera can also be used to find and track a participant wearing colored markers (as was done in an earlier set of experiments we performed). A speaker produces pre-recorded or synthesized speech and sound effects. The motion capture unit provides movement data to the robot wirelessly in real time. The robot control software was implemented using the Player robot control system [20].

We focused on studying how different robot behaviors may affect the patient's willingness to comply with the rehabilitation program. Specifically, we tested different voices, movements, and levels of patience on the part of the robot, and correlated those with participant compliance, i.e., adherence to the exercises. In addition to collecting data about the participant's movement, the human-robot interaction, and task compliance, we also conducted exit interviews and had all participants fill out questionnaires about their impressions of the robot. Finally, all experiments were video-recorded for subsequent analysis.

#### Experiment

The robot system was evaluated in three sessions, each of which featured two subjects. The first session was performed with a non-patient user, while the other two sessions were conducted with stroke survivors. All sessions took place in rehabilitation research labs at the University of Southern California Health Sciences campus. Of the six stroke patients, two were women; all were middle-aged. The stroke impairment occurred on different limbs among the patients but all were sufficiently mobile to perform the activities in the experiments.

**Figure 4:**
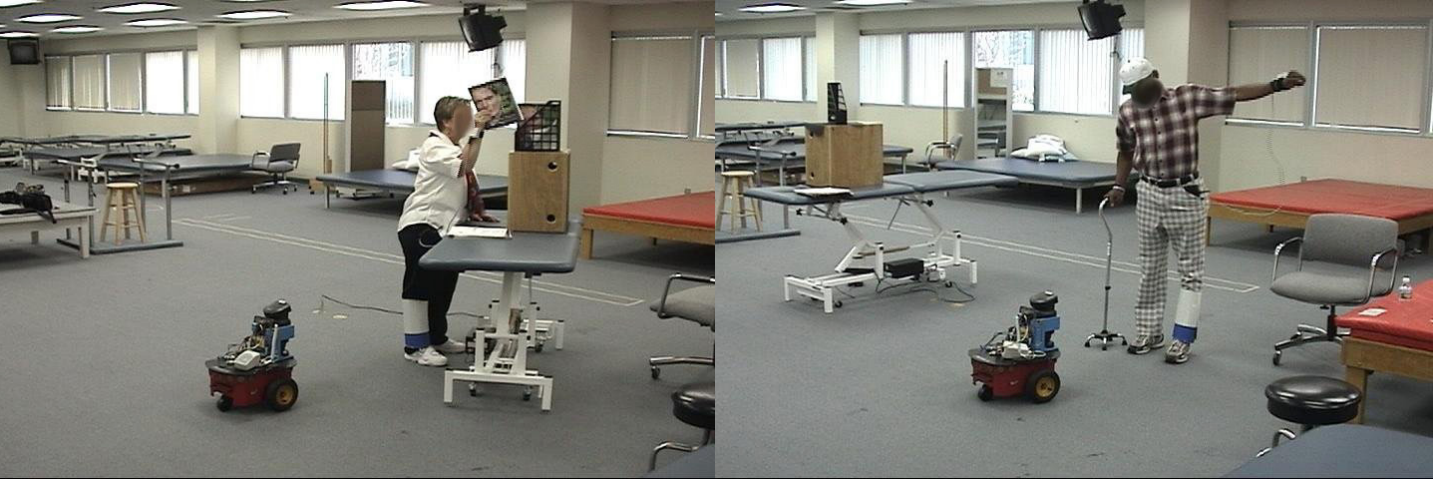
**The two rehabilitation tasks: magazine stacking and free movement of the stroke-affected limb**. The robot and participants during the two rehabilitation experiments: magazine stacking (left) and free movement exercises (right).

The experiments lasted about one hour per person. Every evaluation session comprised six experimental runs. Thus, a total of 36 experiments were performed. In all experiments, the robot asked the participant to perform one of two activities. The first activity was to shelve magazines; its difficulty could be adjusted by using magazines with different weights and varying the height of the bookshelf (Figure 4, left). The robot used the arm motion capture data to determine whether the activity was being performed. Since the robot only received data about the movement of the arm, and not the load on the arm, it was possible to fool the robot by raising the arm without holding a magazine; one patient so fooled the robot (and enjoyed this as an entertaining game). To get around this, the number of magazines shelved was used as the final validation. The second activity consisted of any voluntary activity that involved the movement of the affected arm (Figure 4, right). Here, the robot measured arm movement as an averaged derivative of the arm angle. The compliance measure used in this condition was the total time during which the participant performed the activity.

The robot recognized participant arm movements using the motion capture mechanism described above (see Figure 2), and employed a simple model based on the angle between the arm and the normal to the floor as an indicator of reaching. Figure 2 shows when reaching was detected and when prompting and encouragement were triggered in its absence.

As noted above, three experiments were performed by each participant and for each activity. In each experiment, a different human-robot interaction mode was tested. These modes can be thought of as different robot personalities. The modes used were:

1. The robot gives feedback only through sound effects.

2. The robot uses a synthesized voice and is not persistent.

3. The robot uses a pre-recorded human voice and is persistent.

Sound effects included beeps and pings in response to patient movement. Persistence referred to repeated linguistic prompts and encouragement. The non-persistent robot prompted or encouraged the participant only once in response to a given situation, while the persistent robot did so repeatedly.

For the pre-recorded human voice, we used a female voice in some trials, and a male voice in others, but the content and affect of the pre-recorded speech was kept as identical as possible for both genders.

Our hypothesis was as follows:

*More animated/engaging and persistent robot behavior will result in better patient compliance with the robot's instructions and higher patient approval of the robot*.

#### Results

The questionnaire data conclusively showed that the robot was well-received by both patients and physical therapists. The patients stated that they enjoyed the robot's presence and interactions with it. More enthusiastic interaction modes received higher approval scores. Furthermore, patient compliance with the rehabilitation routine was much higher during the experiments with the robot than under the control (no-robot, no prompting) condition. Both of these results support our hypothesis.

The most prominent feature of the robot personality was the voice it used. Male participants generally preferred the female voices, and vice versa. Interestingly, this is in contrast to work in Human-Computer Interaction (HCI) that showed users consistently preferring a male over female voice in a non-assistive setting [21], illustrating how the assistive setting presents an entirely novel set of biases and challenges for HRI research.

Regardless of the gender of the pre-recorded voice used, all participants preferred the pre-recorded voice to the synthesized voice. This result has important implications for socially assistive robotics, because the use of pre-recorded speech, while technologically simple, does not allow for as much versatility in dialog as does synthesized speech. Our work continues to explore these differences.

One might wonder how the novelty of the robot impacts our results. There is no question that the novelty of the technology had an engaging effect on some subjects. However, since the experiments were quite long in duration (about an hour per participant), the novelty of the experiment can be assumed to have diminished or had been entirely eliminated over time. Importantly, we found that participant behavior relative to the robot did not change over time: participants were either engaged with the robot during the entire trial, or were responsive and compliant but not as actively engaged.

Experiments were terminated after one hour. Some participants continued to do the exercise activity beyond the end of the experiment, but the data were not collected beyond that point. Those participants' data provide further evidence of improved compliance in the robot condition well beyond any novelty effect. The design of the study emphasized the user's response to the robot's behavior. No specific analysis was performed of patient compliance or details of motion capture data. Our subsequent work [15,16] has addressed compliance, and it is a key factor we continue to study actively. Video transcripts of the experiments can be found online [22].

### Embodiment

To address the importance of the robot's physical embodiment and presence in rehabilitative contexts, we designed a follow-up experiment [23]. Three experimental conditions were considered: interaction with a physical robot, interaction with a remote physical robot (through tele-conferencing), and interaction with a virtual robot (simulated using the Gazebo 3D simulator with full dynamics [24]).

#### Experiment

**Figure 5:**
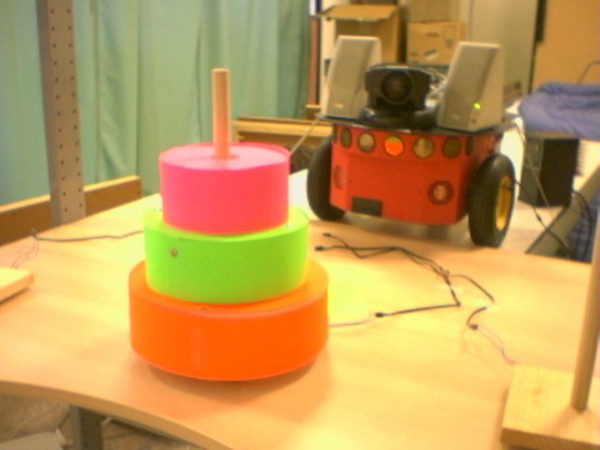
**Tower of Hanoi setup**. The Towers of Hanoi puzzle with three rings and three pegs, as used in the experiments. Also shown is the robot.

**Figure 6:**
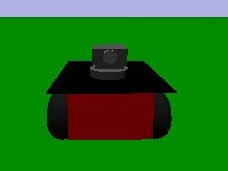
**Simulation of the robot**. The Gazebo 3D simulation with dynamics of a robot used in the embodiment experiment. Shown is the view of the robot as seen by the user.

We designed a task around the classical Towers of Hanoi puzzle, in which rings of different sizes are individually moved from one peg to another (see Figure 5). Since three rings provide relatively few states, the participants quickly grew bored of the game itself and begin to explore the limits of the robot by either breaking the rules of the game or doing nothing to see how the robot would react. The system is sufficiently robust to catch errors and explain to the user how to put the puzzle back into the correct legal state. The three different experimental conditions we tested were:

(a) Physical human-robot interaction: The robot is physically co-located with the participant, by being placed on the table in front of the participant.

(b) Remote presence interaction: The robot is located in another room and its behavior is shown to the participant on a computer screen via a real-time tele-conferencing system.

(c) Simulation interaction: The same screen and audio setup as in (b) but using a 3D simulated virtual robot (see Figure 6) rather than a physical one.

#### Results

Our hypothesis was that the participants would find the physically present robot to be the most watchful and enjoyable of the three conditions. The results from the pilot study support the hypothesis; we are currently conducting a larger follow-up study to expand on those results.

## Discussion

The field of socially assistive robotics is in its inception. As such, there has been little other research done in this area to which our results can be compared. In this section we discuss the experimental results relative to the personality and embodiment components of the above taxonomy, and our ongoing work toward addressing other key challenges of socially assistive robotics.

We noted that some robot personalities during the post-stroke study inspired the subjects to explore and deviate from prescribed behavior. Some modes of interaction were received with interest and even joy, while others were not. Interestingly, however, the less engaging interaction modes let some patients to explore the robot's capabilities. In one case, a participant lead the robot around the room and even outside and down a long corridor, exploring its responses in new situations.

Furthermore, as expected, there were significant personality differences among the participants; some were highly responsive to the robot's prompts but appeared unengaged by the robot, while others were highly engaged and even entertained (as in the above mentioned case), but got involved with playing with the robot rather than performing the prescribed exercises. This leads toward interesting research questions about proper design of adaptive robot-assisted rehabilitation protocols that will serve the variety of patients as well as the time-extended and evolving needs of a single patient. One of our current areas of research involves assessing the participant's personality with a pre-experiment questionnaire, and using the results to adjust the robot's programmed interaction modes. This allows us to study the effectiveness of personality matching, which is known to play a role in human social interactions [15,16].

Unlike non-embodied technologies, robotics allows personality to be expressed not only through voice, facial expressions, and appearance, but also through physical interaction involving movement as a means of capturing and directing user attention and behavior, and the use of personal and social space (proxemics). Our pilot study showed that users had the perception that a robot was more watchful and more enjoyable than an agent on a screen. We are in the process of designing experiments that test whether a user will also have a greater performance on a given task when moderated by a robot than by a computer agent. To address the issue of novelty and lasting effectiveness, we will be conducting time-extended studies with stroke survivors. As discussed in [25], involvement with a social robot decreased after several successive weeks of exposure, but no studies to date have addressed rehabilitative or task-driven interactions with specific rehabilitative goals as are necessary in stroke rehabilitation.

Our continuing experiments are elaborating on the above studies to obtain a significant body of data that addresses the general question of the role of the physical robot embodiment in the hands-off rehabilitation context. We are also designing experimental studies that will further explore the nature of user modeling and interaction by examining physiological measurements for modeling user stress and frustration during therapy.

## Conclusion

Our work is motivated by the potential for significant therapeutic benefit from non-contact human-robot interaction in the context of post-stroke rehabilitation. We have described pilot studies with stroke survivors that support the hypothesis that socially assistive robots are well received by stroke survivors and have a positive impact on their willingness to perform prescribed rehabilitation exercises. Our second pilot study also showed that, while there is a more enthusiastic response to a video of a robot on a screen than a simulation of a robot, there is an even greater response to a physically embodied and co-located robot. Brought together, these results form the basis for our continuing research into non-contact socially assistive robotics in post-stroke and other rehabilitative settings. The goal of socially assistive robotics is to augment human care and existing robot-assisted hands-on therapy toward both improving recovery and health outcomes and making the therapeutic process more enjoyable.
